# Total perfusion-diffusion mismatch detected using resting-state functional MRI

**DOI:** 10.1259/bjrcr.20210056

**Published:** 2021-05-14

**Authors:** Ahmed Khalil, Kian Röhrs, Christian H Nolte, Ivana Galinovic

**Affiliations:** 1 Center for Stroke Research Berlin, Charité Universitätsmedizin, Berlin, Germany; 2 Berlin Institute of Health, Berlin, Germany; 3 Berlin School of Mind and Brain, Humboldt-Universitaet zu Berlin, Berlin, Germany; 4 Department of Neurology, Max Planck Institute for Human Cognitive and Brain Sciences, Leipzig, Germany; 5 Department of Neurology, Charité Universitätsmedizin, Berlin, Germany

## Abstract

Total perfusion-diffusion mismatch is a well-recognised phenomenon in patients with acute ischaemic stroke. We describe a case of total perfusion-diffusion mismatch detected using an emerging contrast-agent-free perfusion imaging technique in a young patient with acute cerebellar stroke.

## Clinical presentation

A 25-year-old male presented 12 min after sudden onset unsteadiness, reporting that he had fallen to the left side three times and experienced neck pain bilaterally and trouble moving his tongue. On examination, the patient had bilateral upper limb dysmetria and bilateral hypermetric ocular saccades (both more pronounced on the left side), was markedly unsteady while standing, and had gait ataxia (National Institutes of Health Stroke Scale [NIHSS]=2). No nystagmus or skew deviation was observed. Stroke was suspected based on the cerebellar signs and confirmed using MRI.

## Investigations/Imaging findings

**Figure 1.**
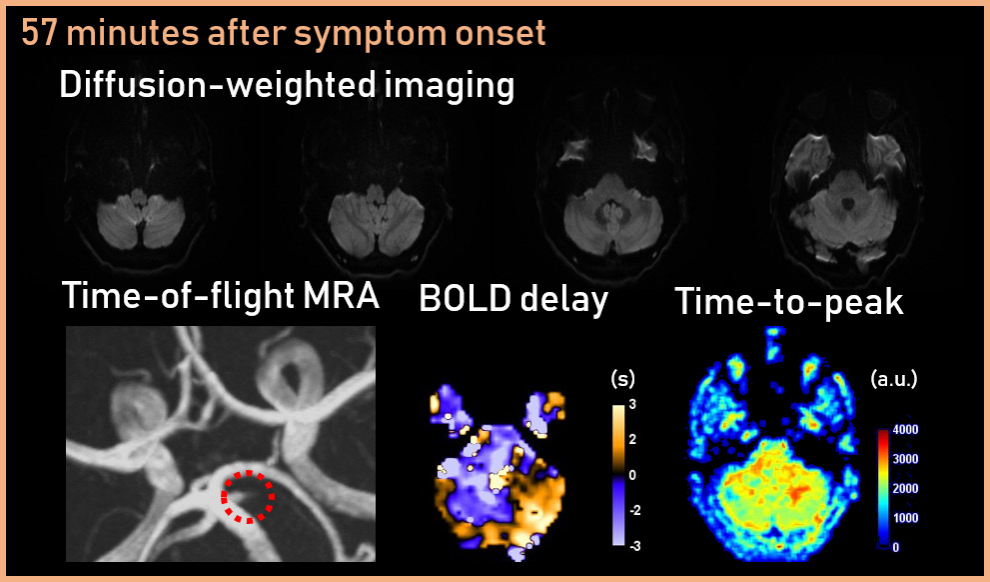
Baseline MRI showed no signs of intracranial haemorrhage or intracerebral restricted diffusion. Time-of-flight magnetic resonance angiography (TOF-MRA) showed left superior cerebellar artery (SCA) occlusion. The BOLD delay and time-to-peak maps showed an area of delayed blood flow in the portion of the left cerebellar hemisphere supplied by the SCA.

A magnetic resonance imaging (MRI) stroke protocol performed 57 min after symptom onset showed no signs of intracranial haemorrhage or intracerebral restricted diffusion (Figure 1). An occlusion of the left superior cerebellar artery (SCA) was seen (matching in location to a thrombus-sign on T2*-weighted imaging, not shown). The blood-oxygenation-level-dependent (BOLD) delay map, derived from time shift analysis of a resting-state functional MRI acquisition (without contrast agent administration)^1^, showed an area of delayed blood flow in the portion of the left cerebellar hemisphere supplied by the SCA. The reference time course for the time shift analysis was derived from the major venous sinuses. The time-to-peak map, derived from a dynamic susceptibility contrast MRI (DSC-MRI) acquisition (after administration of a bolus of Gadobutrol) also showed a discrete, focal area of delayed blood flow in the left SCA territory. Based on clinical presentation and MRI, the patient was diagnosed with acute ischaemic stroke with total perfusion-diffusion mismatch^2^.

## Treatment

The patient was treated with i.v. tissue plasminogen activator 71 min after symptom onset.

### Outcome and follow-up

**Figure 2.**
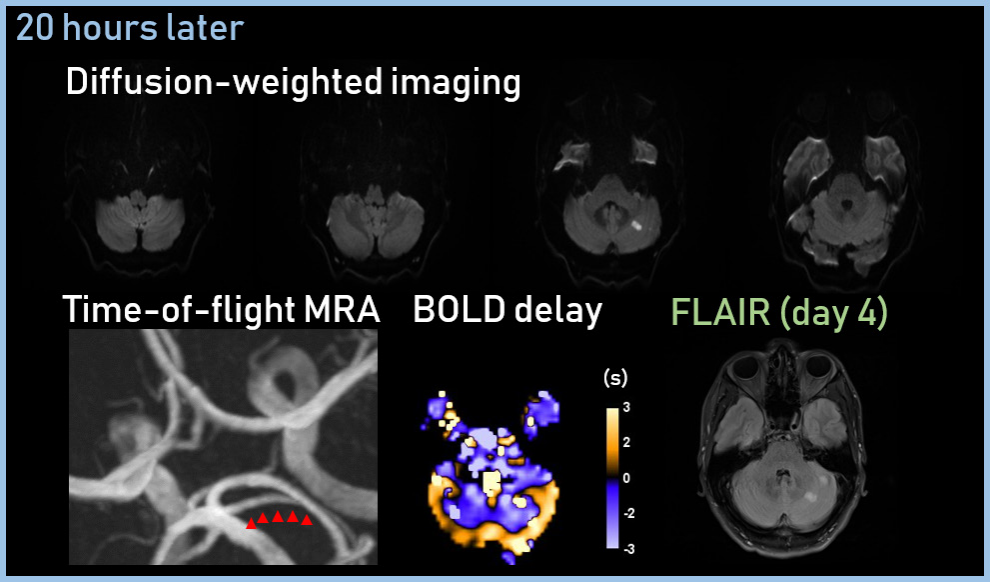
Follow-up MRI showed scattered focal areas of diffusion restriction in the left cerebellar hemisphere, recanalisation of the left SCA, and complete reperfusion in the SCA vascular territory.

The patient received a follow-up MRI 20 h after the initial scan (Figure 2). The follow-up diffusion-weighted image (DWI) showed scattered focal areas of diffusion restriction in the left cerebellar hemisphere, corresponding to the area of the initial perfusion deficit. The follow-up TOF-MRA showed recanalisation of the left SCA and complete reperfusion in the corresponding vascular territory on the BOLD delay map. Follow-up imaging showed no evidence of intracranial haemorrhage after thrombolysis or evidence of vessel dissection.

Further investigations on the stroke unit included routine blood tests, colour Doppler ultrasound of the extracranial arteries, 24 h Holter monitoring, echocardiogram and a vasculitis laboratory work-up, all of which were unremarkable, except for mildly elevated low-density lipoprotein. He was discharged on the sixth day with a diagnosis of cryptogenic stroke, on aspirin and atorvastatin and without any residual complaints or neurological deficits.

### Diagnosis

Cryptogenic acute ischaemic stroke associated with superior cerebellar artery occlusion and complete perfusion-diffusion mismatch.

## Discussion

The most sensitive imaging technique for detecting acute ischaemic stroke is diffusion-weighted imaging (DWI), which shows areas of diffusion restriction reflecting ischaemia-induced cytotoxic oedema. However, like the patient in this study, almost 7% of acute ischaemic stroke patients have negative initial DWI scans.^3^ Several factors are likely to have contributed to the negative initial DWI in this patient, including the fact that MRI was performed less than an hour after stroke onset and that the stroke was in the posterior circulation.^3^

Often, diffusion restriction on DWI overlaps with, or is surrounded by, an area of low or delayed blood flow, referred to as a perfusion deficit. “Total perfusion-diffusion mismatch” is a well-recognised imaging pattern describing the presence of a perfusion deficit without a corresponding diffusion restriction and is seen in between 0.05 and 3% of all acute ischaemic stroke patients.^2,4,5^ Importantly, in the setting of total mismatch, evidence of tissue ischaemia on DWI often appears with time,^6^ as was the case in this patient. This highlights the importance of the added information provided by perfusion imaging in the workup of patients suspected of having acute ischaemic stroke. Such additional information is particularly useful when stroke mimics cannot be reliably excluded or the clinical presentation is unclear.

Several established methods for MRI-based perfusion imaging exist. DSC-MRI is the most commonly used, but it requires the injection of gadolinium-based contrast agents, which should be used with caution in patients with impaired renal function.^7^ Additionally, the repeated use of gadolinium-based contrast agents is discouraged due to their potential for accumulating in the brain,^8^ making DSC-MRI a suboptimal choice for longitudinal perfusion monitoring.

Recently, techniques such as BOLD delay have garnered interest ^9,10^ because they do not require exogenous contrast agent administration, despite being more sensitive to patient motion than DSC-MRI. As illustrated in this case, BOLD delay is capable of demonstrating total perfusion-diffusion mismatch and monitoring perfusion changes over time in a non-invasive manner.

## Learning points

- Diagnosis of posterior fossa infarcts in emergency care settings is challenging, especially in the young.
- Normal DWI despite persistent, acute-onset central neurological symptoms and evidence of vessel occlusion should raise suspicion of a total perfusion-diffusion mismatch.
- Non-invasive perfusion imaging based on resting-state functional MRI (“BOLD delay”) is capable of detecting total perfusion-diffusion mismatch and may be used in situations where it is suspected. This may be particularly useful in patients with impaired renal function (where gadolinium-based contrast agents are contraindicated).
